# Dalbergiones lower the inflammatory response in oral cells in vitro

**DOI:** 10.1007/s00784-022-04509-7

**Published:** 2022-05-04

**Authors:** Feng Shao, Layla Panahipour, Anes Omerbasic, Fangrui Tang, Reinhard Gruber

**Affiliations:** 1 Department of Oral Biology, Medical University of Vienna, Sensengasse 2a, 1090 Vienna, Austria; 2 Key Laboratory of Modern Preparation of Traditional Chinese Medicine, Ministry of Education, Jiangxi University of Chinese Medicine, Meiling Road 1688, 330004 Nanchang, China; 3 Department of Periodontology, School of Dental Medicine, University of Bern, Freiburgstrasse 7, 3010 Bern, Switzerland; 4 Austrian Cluster for Tissue Regeneration, Donaueschingenstraße 13, 1200 Vienna, Austria

**Keywords:** 3’-Hydroxy-4,4’-dimethoxydalbergione, 4-Methoxydalbergione, 4’-Hydroxy-4-methoxydalbergione, Anti-inflammatory activity, Periodontitis, RAW 264.7 macrophages, Gingival fibroblasts

## Abstract

**Objectives:**

Periodontitis is a global health burden that underlines the demand for anti-inflammatory treatment. *Dalbergia melanoxylon* being a rich source of flavonoids has been widely used in traditional medicine but the potential anti-inflammatory activity of its dalbergiones remains to be shown.

**Material and methods:**

We have isolated 3′-hydroxy-4,4′-dimethoxydalbergione, 4-methoxydalbergione, and 4′-hydroxy-4-methoxydalbergione from *Dalbergia melanoxylon* and tested their potential anti-inflammatory activity.

**Results:**

All dalbergiones are potent inhibitors of an LPS-induced inflammatory response of RAW 264.7 macrophages. This is specified by IL1β and IL6 production, and the p65 nuclear translocation. Consistently, in primary macrophages, the dalbergiones caused an M1-to-M2 polarization switch indicated by the decreased ration of IL1β and IL6 versus arginase 1 and YM1 expression. To implement oral cells, we have used gingival fibroblasts exposed to IL1β and TNFα. Consistently, all dalbergiones reduced the expression of IL6 and IL8 as well as the nuclear translocation of p65.

**Conclusion:**

These findings increase the accumulating knowledge on dalbergiones and extend it towards its capacity to lower the inflammatory response of oral cells.

**Clinical relevance:**

These findings are another piece of evidence that supports the use of herbal medicine to potentially lower inflammatory events related to dentistry.

## Introduction

Periodontitis and other forms of chronic inflammation such as periimplantitis and mucositis occurring in the oral cavity are pathological conditions that when exceeding culminate in local tissue destruction and tooth or implant loss [1, 2]. The major causes of chronic inflammation are virulence factors originating from dental plaque, thus mainly bacteria forming a biofilm [1]. It is thus of clinical importance to remove the biofilm allowing the natural process of resolving the inflammation and to regain a healthy physiological tissue homeostasis [1]. However, clinical strategies based on the mechanical removal of biofilm are not only supported by the application of local delivery of antibiotics and antimicrobials such as chlorhexidine [3] and systemic antibiotics [4]. Alternative or even synergistic strategies focus towards the local application of natural anti-inflammatory compounds such as curcumin [5] that ideally also support the resolution of inflammation [6]. Nature developed a large spectrum of candidate molecules equipped with an anti-inflammatory capacity supporting the success of the traditional herbal medicine [7]. The WHO advocates the inclusion of herbal medicine in primary health.

Dalbergiones are naturally occurring anti-inflammatory compounds originating from *Dalbergia melanoxylon,* which is a plant widespread in tropical and subtropical Africa regions [8]. The generic name *Dalbergia* honors the Swedish brothers, Nils and Carl Dalberg, who lived during the eighteenth century. Aqueous root extract and methanol leaf extract of *D. saxatilis* hold anti-inflammatory properties in rat models [9, 10]. Moreover, ethanol extracts from *D. lanceolaria* bark had anti-inflammatory activity in rodents [11]. Extracts are however a heterogenous mixture of components and it requires isolation to characterize their chemical and biological properties. Resent reviews highlight the phytochemistry and link it to the biological activity of *D. melanoxylon* [12]. Today’s pharmacology research has established that isolated components from the heartwood and bark possess wide-ranging pharmacological effects, including anti-inflammatory activity [13]. *Dalbergia*, however, is highly threatened as a genus globally because of its deforestation and illegal harvesting and in the IUCN Red List of Threatened Species™. There is yet a need for drug discovery research to understand the biology of *D. melanoxylon*.

Among the compounds isolated is 3, 4-dimethoxydalbergione that was originally proposed to cause contact dermatitis to exotic woods [14, 15]. Other compounds isolated from *D. louvelii* including 4-methoxydalbergione (MD) have antiplasmodial activity [16], and inhibited the release of glucuronidase and histamine from isolated rat cells proposing an anti-inflammatory activity [17]. However, there are more compounds with a potential anti-inflammatory activity such as 3′-hydroxy-4,4′-dimethoxydalbergione (HDMD) and 4′-hydroxy-4-methoxydalbergione (DMD) that have not been evaluated so far. Moreover, the role of dalbergiones to reduce the response of cells relevant for oral inflammatory diseases such as macrophages and fibroblasts of the gingiva has not been tested in vitro. We know that MD can increase heme oxygenase (HO) 1 levels in microglial cells and thereby suppress LPS-stimulated inflammatory response and nuclear factor-kappa B (NF-κB) signaling [18]. Similar studies with respect to other dalbergiones and focusing on oral inflammation have not been performed.

Here, we provide data supporting our conclusion that not only MD but also HDMD and DMD are potent inhibitors of an inflammatory response of macrophages even causing an M1-to-M2 polarization switch and gingival fibroblasts in vitro thereby helping to identify natural anti-inflammatory compounds and supporting their use in herbal medicine that extends toward dentistry.

## Methods

### Extraction of compounds of *Dalbergia melanoxylon*

The isolation of the three compounds was reported in detail [19]. In brief, the heartwood of *D. melanoxylon* was purchased from Fang Chenggang market, Guangxi Province, China in July 2014 and identified by the product quality inspection center of Guangxi University in Guangxi Province, China. A voucher specimen (No. Liu-20140713) was deposited in the Key Laboratory of Innovation Drug and Efficient Energy Saving Pharmaceutical Equipment, Jiangxi University of Chinese Medicine. The air-dried pieces of heartwood of *D. melanoxylon* (50.0 kg) were powdered, passed through a #40 mesh sieve, and extracted with 70% ethanol under refluxing three times. After removing the solvent under reduced pressure, the residue (13.9 kg) was suspended in water and partitioned with dichloromethane, ethyl acetate, and n-butanol, respectively.

The dichloromethane extract (8.5 kg) was subjected to silica gel column chromatography. A petroleum ether/EtOAc gradient yielded 22 fractions (Frs.1–16). Frs.7 (447.4 g) and was further separated using a CH_2_Cl_2_/MeOH gradient to obtain Frs.7.A—Frs.7.K. Frs.7.C (1.2 g) was subjected to Sephadex LH-20 chromatography eluted with CH_2_Cl_2_/MeOH (1:1) to isolate MD (16.2 mg). Frs.8 (147.4 g) was further separated into 3 fractions (Frs.8.1–3) and subjected to a gradient of CH_2_Cl_2_/MeOH. Frs.8.1 (120.1 mg) was further separated by a semi-preparative HPLC eluted with ACN/H_2_O (60:40) to isolate HDMD (6.1 mg). The chemical structure of MD and HDMD was confirmed by nuclear magnetic resonance spectroscopy for HDMD [20] and MD [21]. MD and HDMD were dissolved in dimethyl sulfoxide and stored at frozen.

The ethyl acetate extract (1.8 kg) was separated into 12 fractions (Frs.1–12) by silica gel column chromatography and eluted with a CH_2_Cl_2_/MeOH gradient. Frs.7 (15.7 g) was separated by a CH_2_Cl_2_/MeOH gradient to obtain Frs.7.A-Frs.7.H. The Frs.7.D (1.4 g) was then separated into 6 subfractions (Frs.7.D.1–6) by a CH_2_Cl_2_/MeOH gradient. Frs.7.D.3 (303.8 mg) was again separated into 7 subfractions (Frs.7.D.3.A-G) and eluted gradient with a petroleum ether/EtOAc gradient. Frs.7.D.3.F (114.0 mg) was further separated by a semi-preparative HPLC and eluted with MeOH/H_2_O (55:45) to isolate DMD (23.4 mg). The chemical structure of DMD was confirmed by nuclear magnetic resonance spectroscopy [22]. DMD was dissolved in dimethyl sulfoxide and stored in aliquots until being used for in vitro testing.

### Murine bone marrow macrophages, human gingival fibroblasts, and cell lines

BALB/c mice at the age of 6–8 weeks were purchased from Animal Research Laboratories, Himberg, Austria. The femora and tibiae of the mice were removed after scarifying and bone marrow cells were collected. Cells were seeded at 3 × 10^6^cells/cm^2^ into 12-well plates and grown for 5–7 days in Dulbecco’s Modified Eagle Medium supplemented with 10% fetal calf serum, 1% of 10,000 units penicillin and 10 mg/mL streptomycin. (Sigma, St Louis, MO, USA) and with 20 ng/mL mouse macrophage colony-stimulating factor (M-CSF; ProSpec-Tany TechnoGene Ltd., Rehovot, Israel). RAW 264.7 macrophage-like cells (ATCC; LGC Standards GmbH, Wesel, Germany) were expanded in regular DMEM growth medium without supplement. Human gingival fibroblasts were prepared from explant culture after approval of the Ethical Committee of the Medical University of Vienna (EK Nr. 631/2007). All cells were cultured under standard conditions at 37 °C, 5% CO_2_, and 95% humidity.

### Cell stimulation

Primary macrophages and RAW 264.7 cells were exposed to 20 µM HDMD, MD, and DMD for 30 min before adding 100 ng/mL LPS for 24 h. Gingival fibroblasts were exposed to dalbergiones for 30 min before adding IL1β and TNFα (both at 10 ng/mL, ProSpec-Tany TechnoGene Ltd., Rehovot, Israel) in serum-free medium. After 24 h, gene expression analysis was performed and the supernatant was collected for immunoassay.

### RT-PCR and immunoassay

Total RNA was isolated with the ExtractMe total RNA kit (Blirt S.A., Gdańsk, Poland). Reverse transcription was performed with SensiFAST cDNA kit (Bioline, London, UK). Polymerase chain reaction was done with the SensiFAST master mix (Bioline). Amplification was monitored with the CFX Connect™ Real-Time PCR Detection System (Bio-Rad Laboratories, CA, USA). Primer sequences are provided in Table 1. The mRNA levels were calculated by normalization to the housekeeping gene GAPDH and actin using the ΔΔCt method. For the immunoassay, the mouse and human IL6/IL8 kit was used (R&D Systems, Minneapolis, MN, USA).

**Table 1:** The primer sequences

Primers	Sequence_F	Sequence_R
mIL	AAGGGCTGCTTCCAAACCTTTGAC	ATACTGCCTGCCTGAAGCTCTTGT
mIL6	GCTACCAAACTGGATATAATCAGGA	CCAGGTAGCTATGGTACTCCAGAA
mCox2	CAGACAACATAAAACTGCGCCTT	GATACACCTCTCCACCAATGACC
mArg1	GAATCTGCATGGGCAACC	GAATCCTGGTACATCTGGGAAC
mYm1	GGGCATACCTTTATCCTGAG	CCACTGAAGTCATCCATGT
mGAPDH	AACTTTGGCATTGTCGAACG	GGATGCAGGGATGATGTTCT
hIL6	GAAAGGAGACATGTAACAAGAGT	GATTTTCACCAGGCAAGTCT
hIL8	AACTTCTCCACAACCCTCTG	TTGGCAGC CTTCCTGATTTC
hGAPDH	AAGCCACATCGCTC AGACAC	GCCCAATACGACCAAATCC

### Western blot

RAW 264.7 cells and gingival fibroblasts were serum-starved overnight and then preincubated for 10 min with dalbergiones before being exposed for 25 min to LPS (100 ng/mL). Cell extracts containing SDS buffer and protease inhibitors (PhosSTOP with complete; Sigma, St. Louis, MO, USA) were separated by SDS-PAGE and transferred onto nitrocellulose membranes (Whatman, GE Healthcare, General Electric Company, Fairfield, CT, USA). Membranes were blocked and the binding of the first antibody raised against phospho-NF-κB p65 and NF-κB p65 (#3033 and #8242; Cell Signaling Technology, Danvers, MA, USA) and beta-actin (Santa Cruz Biotechnology, Santa Cruz, CA, USA) was detected with the appropriate secondary antibody linked to a peroxidase. Chemiluminescence signals were visualized with the ChemiDoc imaging system (Bio-Rad Laboratories, Inc., Hercules, CA, USA).

### Immunofluorescence

The immunofluorescent analysis of p65 was performed in RAW 264.7 cells and gingival fibroblasts plated onto Millicell® EZ slides (Merck KGaA, Darmstadt, Germany). Serum-starved cells were exposed to dalbergiones for 10 min following the inflammatory response provoked by LPS, IL1β, and TNFα for 60 min, respectively. The cells were fixed with 4% paraformaldehyde, blocked with 1% bovine serum albumin, and permeabilized with 0.3% Triton. We used rabbit anti-human NFκB p65 (#8242; Cell Signaling Technology, Cambridge, UK) at 4 °C overnight. Detection was performed with the goat anti-rabbit Alexa 488 secondary antibody (CS-4412, Cell Signaling Technology). Images were captured under a fluorescent microscope with a single filter block 455 nm (Oxion fluorescence, Euromex, Arnheim, Netherlands).

### Statistical analysis

All experiments were repeated at least three times. Data from individual experiments are shown as dot-blots. Data are described as x-fold change compared to unstimulated control. Statistical analyses were based on Friedman test for nonparametric but paired samples. Data were analyzed by the Prism 8.0e software (GraphPad Software; San Diego, CA, USA).

## Results

### Isolation of 4-methoxydalbergione (MD) and 3′-hydroxy-4,4′-dimethoxydalbergione (HDMD)

**Fig. 1:**
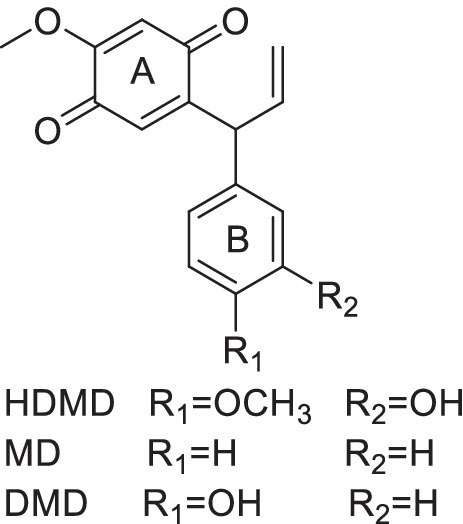
The structure of dalbergiones

To isolate chemically defined natural anti-inflammatory compounds, we reconstituted the ethanolic extracts of *D. melanoxylon *heartwood with dichloromethane and ethyl acetate. The dichloromethane solution was used to isolate 16.2 mg MD and 6.1 mg HDMD. The ethyl acetate solution provided 23.4 mg DMD. The chemical structure of the three isolated and purified dalbergiones was confirmed by nuclear magnetic resonance spectroscopy and is indicated in Fig. 1. The ethanolic extracts of the heartwood of *D. melanoxylon* were resuspended in dichloromethane extract and fractionated by chromatography and HPLC to isolate HDMD and MD. The ethyl acetate extracts were repeatedly subjected to chromatography and HPLC to isolate DMD. The chemical structure was confirmed by nuclear magnetic resonance spectroscopy.

### HDMD, MD, DMD cause an M1-to-M2 shift in macrophage polarization

**Fig. 2:**
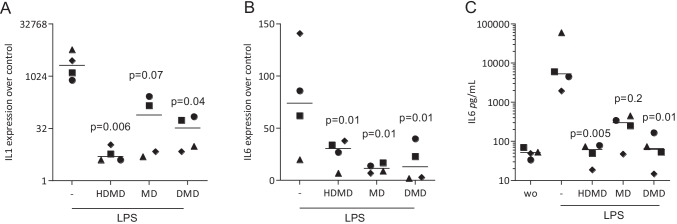
HDMD, MD, and DMD suppress LPS-induced expression of cytokines in RAW 264.7 cells. RAW 264.7 cells were exposed to 20 µM of HDMD, MD, and DMD for 30 min before cells were treated with 100 ng/mL LPS for 24 h. Data show x-fold changes in (**A**) IL1β and (**B**) IL6 expression compared to unstimulated cells. (**C**) The immunoassay shows the release of IL6 into the supernatant of the respective cultures. *N* = 4. Statistical analysis was based on Friedman test, and *P*-values are indicated compared to the LPS group. Significance was set at *P* < 0.05

**Fig. 3:**
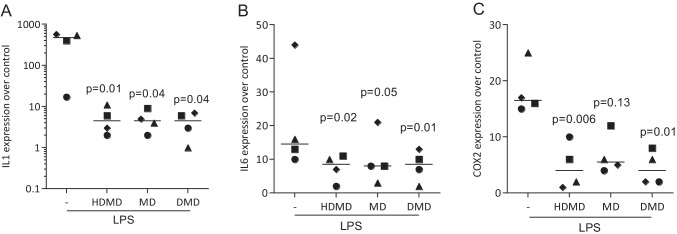
HDMD, MD, and DMD suppress LPS-induced expression of M1 marker cytokines in primary macrophages. Primary macrophages were exposed to 20 µM of HDMD, MD, and DMD for 30 min before cells were treated with 100 ng/mL LPS for 24 h. Data show the x-fold changes in (**A**) IL1β, (**B**) IL6, and (**C**) COX2 expression compared to unstimulated cells. *N* = 4. Statistical analysis was based on Friedman test, and *P*-values are indicated compared to the LPS group. Significance was set at *P* < 0.05

**Fig. 4:**
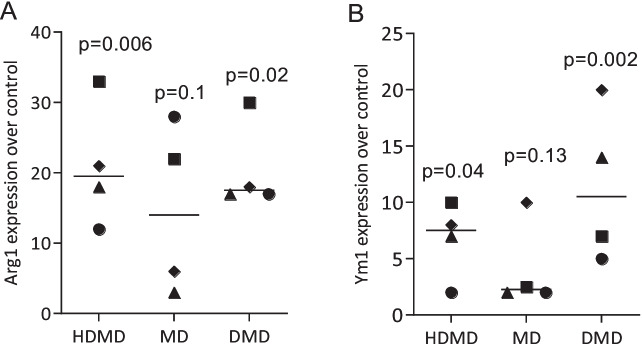
HDMD, MD, DMD can increase the expression of M2 marker genes in primary macrophages. Primary macrophages were exposed to 20 µM of HDMD, MD, and DMD for 24 h. Data show the x-fold changes in (**A**) Arg1 and (**B**) Ym1 expression compared to unstimulated cells. *N* = 4. Statistical analysis was based on Friedman test. Significance was set at *P* < 0.05

First, we have done a dose–response formazan toxicity assay with RAW 264.7 cells suggesting that 100 µM but not 20 µM HDMD, MD and DMD is toxic (data not shown). Next and to identify the possible anti-inflammatory activity, we have preexposed RAW 264.7 and primary macrophages to the three dalbergiones before provoking an M1 inflammatory response with LPS [23, 24]. We report there that the robust increase of the cytokines IL1 and IL6 caused by LPS is almost completely abolished when RAW 264.7 cells (Fig. 2) and primary macrophages (Fig. 3) were exposed to 20 µM of HDMD, MD, and DMD. The strong anti-inflammatory activity of the three dalbergiones was observed on the transcription but also on the translation level as indicated for IL6 (Fig. 2 and Fig. 3). Moreover, in primary macrophages, all three dalbergiones induced the expression of Arg1 and YM1, both marker genes of M2 polarization (Fig. 4). These findings therefore have revealed the potential of HDMD, MD and DMD to cause an M1-to-M2 shift in macrophage polarization.

### HDMD, MD, DMD greatly diminish the p65 nuclear translocation and phosphorylation in macrophages

**Fig. 5:**
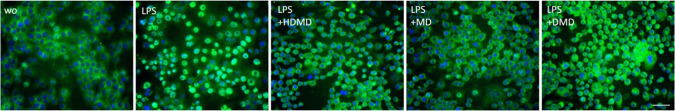
HDMD, MD and DMD attenuate the translocation of NFkB from the cytoplasm into the nucleus. RAW 264.7 cells were exposed to 20 µM of HDMD, MD, and DMD for 10 min before cells were treated with LPS for 1 h. Immunofluorescence analysis shows the intracellular translocation of NFkB p65 into the nucleus in cells stimulated with LPS but not in the untreated cells or in the presence of dalbergiones. The size bar represents 100 µm

**Fig. 6:**
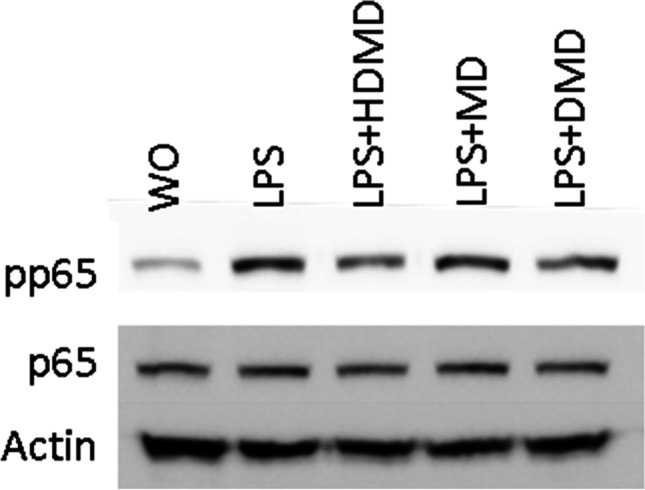
HDMD, MD and DMD reduced the phosphorylation of p65. RAW 264.7 cells were exposed to 20 µM of HDMD, MD, and DMD for 30 min before cells were treated with LPS for 30 min. Western blot analysis shows the phosphorylation of p65 of cells stimulated with LPS but not in the untreated cells or in the presence of dalbergiones

To identify the underlying molecular mechanism, we have tested to which extend the dalbergiones reduce the canonical p65-NFkB signaling pathway in RAW 264.7 macrophages. Immunostaining revealed that all three dalbergiones effectively hindered p65 to translocate into the nucleus (Fig. 5). Moreover, Western blot showed that the exposure of RAW 264.7 macrophages to 20 µM of HDMD, MD, DMD greatly reduced the LPS-induced phosphorylation signal of p65 (Fig. 6). Taken together, the potent anti-inflammatory activity of HDMD, MD, DMD is associated with a blocking of p65-NFkB signaling in vitro.

### HDMD, MD, DMD reduce the inflammatory response of gingival fibroblasts to IL1β and TNFa

**Fig. 7:**
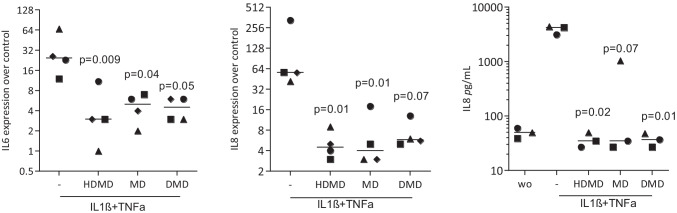
HDMD, MD, and DMD suppress IL1β/TNFα-induced expression of cytokines in gingival fibroblasts. Gingival fibroblasts were exposed to 20 µM of HDMD, MD, and DMD for 30 min before cells were treated with IL1β and TNFα for 24 h. Data show x-fold changes in gene expression compared to unstimulated cells. The immunoassay shows the release of IL8 into the supernatant of the respective cultures. *N* = 4. Statistical analysis was based on Friedman test, and *P* values are indicated compared to the LPS group. Significance was set at *P* < 0.05

**Fig. 8:**
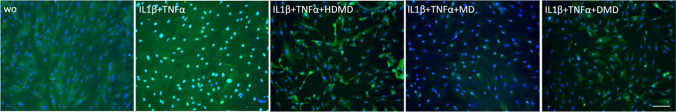
HDMD, MD, and DMD attenuate the translocation of NFkB p65 from the cytoplasm into the nucleus*.* Gingival fibroblasts were exposed to 20 µM of HDMD, MD, and DMD for 10 min before cells were treated with IL1β and TNFα for 1 h. Immunofluorescence analysis shows the intracellular translocation of NFkB p65 into the nucleus in cells stimulated with IL1β and TNFα but not in the untreated cells or in the presence of dalbergiones. The size bar represents 100 µm

To understand if the anti-inflammatory activity is also present in cells of the oral cavity, gingival fibroblasts were treated with dalbergiones before exposing them to IL1β and TNFα. As expected [24, 25] the gingival fibroblasts showed a robust inflammatory response indicated by the expression of IL6 and IL8 (Fig. 7). Consistent with what we have observed with macrophages, HDMD, MD, DMD reduced the inflammatory response of gingival fibroblasts when exposed to IL1β and TNFα (Fig. 7). Immunostaining showed that dalbergiones hindered p65 to translocate into the nucleus (Fig. 8). Taken together, gingival fibroblasts are target cells for HDMD, MD, DMD to exert their potent anti-inflammatory activity.

### HDMD, MD, DMD requires HO1 signaling to exert its anti-inflammatory activity

**Fig. 9:**
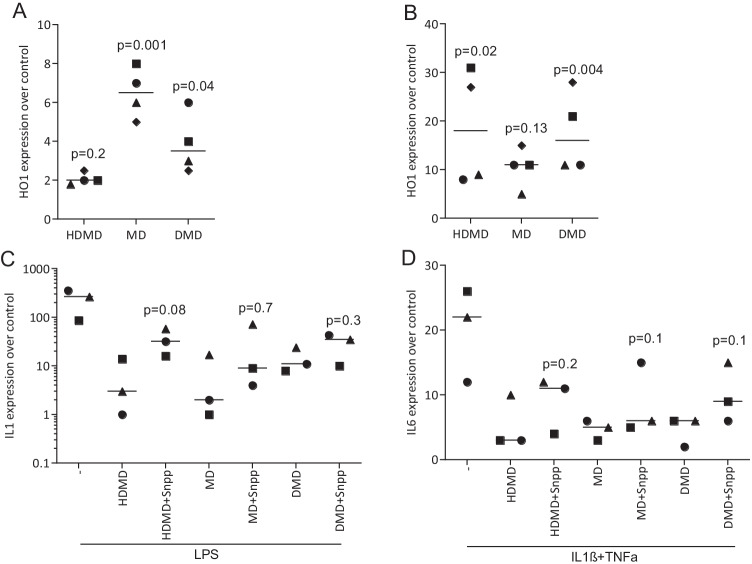
HDMD, MD and DMD increase HO1 expression and its blocking partially reversed their activity. RAW 264.7 macrophages and gingival fibroblasts were exposed to 20 µM of HDMD, MD, and DMD for 24 h with and without the presence of SnPP. Gene expression analysis shows that all three dalbergiones increased HO1 expression in (**A**) RAW 264.7 macrophages and (**B**) gingival fibroblasts. Even though not reaching the level of significance, there is a clear trend that SnPP could at least partially reverse the anti-inflammatory activity of dalbergiones in (**C**) RAW 264.7 macrophages and (**D**) gingival fibroblasts. Symbols represent independent experiments. Statistic is based on a Friedmann test and *P*-values are shown

To determine the involvement of HO1 signaling to mediate the effects of dalbergiones in RAW264.7 macrophages and gingival fibroblasts, SnPP, an inhibitor of HO1 signaling, was introduced. Gene expression analysis showed that HDMD, MD, DMD significantly increased the expression of HO1 in RAW 264.7 cells (Fig. 9A) and gingival fibroblasts (Fig. 9B). SnPP at least partially reversed the anti-inflammatory activity indicated by the expression of IL1β of the three dalbergiones in RAW 264.7 cells (Fig. 9C) and IL6 gingival fibroblasts (Fig. 9D). These observations suggest that HDMD, MD, DMD exert only a small part of their anti-inflammatory activity via the activation of HO1 signaling.

## Discussion

Local chronic inflammation is a hallmark of oral diseases such as periodontitis and periimplantitis that is mainly driven by the accumulation of bacterial plaque that provides a reservoir for the respective virulence factors constantly causing an innate immune response [1]. Apart from the mechanical removal of the plaque, local delivery of antibiotics and antimicrobials [3] and systemic antibiotics [4] are standard treatment in periodontitis. However, it requires the host cells to shift from their katabolic inflammatory stage into an anabolic resolving stage to pave the way for the regeneration and repair of the original periodontal tissue [6]. The local delivery of anti-inflammatory and resolving substances from natural origin, previously implemented in traditional herbal medicine has a great potential to accomplish this therapeutic goal. Based on this concept, we have focused on the isolation as well as the chemical and biological characterization of dalbergiones isolated from *D. melanoxylon*.

The main finding of the present research was that we could isolate three bioactive members of the dalbergiones, namely, HDMD, MD, and DMD, all capable to greatly diminish the LPS and IL1β/TNFα-induced inflammatory response of macrophages and gingival fibroblasts, respectively, in vitro. Moreover, the three dalbergiones caused an M1-to-M2 shift in macrophages, which is an indirect support for a potential resolving activity [26]. Thus, the local application of dalbergiones may support the resolution of inflammation after removal of the dental plaque. Moreover, if we relate the findings to those of others, our observations are in support of MD to suppress the LPS-stimulated inflammatory response in microglial cells involving HO1 and NF-κB signaling [18]. No reports on HDMD and DMD are available so far even though the number and position of substituents in the same fundamental structure of these dalbergiones, such as phenolic hydroxyl group supposes a structure anti-inflammatory activity relationship.

Even though all three dalbergiones have a strong anti-inflammatory potential, one might get the impression that there is a subtle difference in their anti-inflammatory activities. The decreasing order of the inflammatory activity was as follows: HDMD > DMD > MD. There is the same structure in A ring of the three components, but a different substituted group in B ring. It can be hypothesized that the number and position of the methoxyl group and the phenolic hydroxyl group were responsible for their anti-inflammation. Thus, it can be speculated that DMD showed stronger activity than MD probably because of the phenolic hydroxyl group in B ring. This result agreed with the previous report showing that DMD showed stronger activity of preventing NO production by LPS-stimulated J774.1 cells than MD, indicating that the presence of an phenolic hydroxyl group at 4′ affects the inhibitory activity of dalbergiones [13]. Not only that, our results indicated the presence of the methoxyl group in B ring of HDMD could further increase anti-inflammatory activity. It may be caused by the methoxyl group changing hydrophobicity and molecular planarity [27]. However, because of all three dalbergiones are highly effective in suppressing an inflammatory response in vitro, conclusions on a structure-functional relationship should not be drawn.

The study has limitations. Even though dalbergiones possess a robust anti-inflammatory activity in vitro, the underlying mechanisms remain partially unclear. Good evidence for the expected blocking of the NFkB signaling pathway comes from immunostainings presenting suppression of p65 nuclear translocation, and to some extend also from Western blot analysis showing a decreased capacity of LPS to cause phosphorylation of p65 in RAW 264.7 macrophages. However, and even though all three dalbergiones increased HO1 expression in RAW 264.7 and gingival fibroblasts, SnPP-induced blocking of HO1 signaling only moderately reversed the anti-inflammatory activity of HDMD, MD, and DMD in vitro. Thus, it remains unclear to which extend the HO1 signaling pathway mediates the anti-inflammatory activity of the three dalbergiones in macrophages and gingival fibroblasts. Future studies should use macrophages from HO1 or Nrf2 knockout mice to address this research question.

Further studies should also extend our observation that dalbergiones increase M2-macrophage marker genes Arg1 and YM1, providing indirect evidence for the transplanting of cells from an inflammatory towards a resolving phenotype, similar to what was proposed for ginsenosides [28]. However, in vitro bioassay can only partially reflect the complex inflammatory environment in vivo, and to which extend and by which route of application dalbergiones may serve as resolving compounds needs to be determined. There is, however, a reason to suggest a strong potential for the dalbergiones as HO1 induction can drive the phenotypic shift to M2 macrophages, also suggesting HO1 induction in macrophages is a potential therapeutic approach to control inflammation and by that also tissue regeneration [29]. We have to confess that our observation on the strong M1-to-M2 shift in macrophages and the reduced inflammatory response in the gingival fibroblasts provide a primer for a future more translational research, for instance the local injection of the dalbergiones at sites previously exposed to inflammatory clues, in a ligature-induced periodontitis model [30].

Our research revealed the strong and robust anti-inflammatory activity of the three dalbergiones HDMD, MD, and DMD based on established macrophage and fibroblast in vitro bioassays. These outcomes pave the way for future research to better understand the underlying molecular mechanism and further to translate the findings towards preclinical testing in a relevant model of chronic inflammation such a periodontitis being aware that the therapeutic potential of dalbergiones exceeds dentistry, similar to what has been proposed for other compounds in herbal medicine.

## Data Availability

The datasets used are available from the corresponding author on reasonable request.

## Abbreviations

HDMD: 3′-Hydroxy-4,4′-dimethoxydalbergione
MD: 4-Methoxydalbergione
DMD: 4′-Hydroxy-4-methoxydalbergione
HO1: Heme oxygenase 1
Nrf2: Nuclear factor erythroid 2–related factor 2
SnPP: Tin protoporphyrin IX

## References

[1] Kinane DF, Stathopoulou PG, Papapanou PN (2017) Periodontal diseases. Nat Rev Dis Primers 3:17038. 10.1038/nrdp 10.1038/nrdp.2017.38 28805207

[2] Pihlstrom BL, Michalowicz BS, Johnson NW (2005). Periodontal diseases. Lancet.

[3] Hanes PJ, Purvis JP (2003). Local anti-infective therapy: pharmacological agents. A systematic review. Ann Periodontol.

[4] Slots J (2002). Selection of antimicrobial agents in periodontal therapy. J Periodontal Res.

[5] Pérez-Pacheco CG, Fernandes NAR, Primo FL, Tedesco AC, Bellile E, Retamal-Valdes B, Feres M, Guimares-Stabili MR, Rossa C (2020). Local application of curcumin-loaded nanoparticles as an adjunct to scaling and root planing in periodontitis: randomized, placebo-controlled, double-blind split-mouth clinical trial. Clin Oral Investig.

[6] Van Dyke TE (2020). Shifting the paradigm from inhibitors of inflammation to resolvers of inflammation in periodontitis. J Periodontol.

[7] Wei X, Zhao ZJ, Zhong RH, Tan X (2021). A comprehensive review of herbacetin: from chemistry to pharmacological activities. J Ethnopharmacol.

[8] Jenkins M, Oldfield SF, Aylett T (2002). International trade in African blackwood.

[9] Ismail HF, Zezi AU, Hamza YA, Habib DU (2015). Analgesic, anti-inflammatory and antipyretic activities of the methanol leaf extract of *Dalbergia saxatilis* Hook.F in rats and mice. J Ethnopharmacol.

[10] Yemitan OK, Adeyemi OO (2017). Mechanistic assessment of the analgesic, anti-inflammatory and antipyretic actions of *Dalbergia saxatilis* in animal models. Pharm Biol.

[11] Kale M, Misar AV, Dave V, Joshi M, Mujumdar AM (2007). Anti-inflammatory activity of *Dalbergia lanceolaria* bark ethanol extract in mice and rats. J Ethnopharmacol.

[12] Najeeb TM, Issa TO, Mohamed YS, Ahmed RH, Maknawi AM, Khider TO (2018). Phytochemical Screening, antioxidant and antimicrobial activities of Dalberegia melanoxylon tree. World Appl Sci J.

[13] Shrestha SP, Amano Y, Narukawa Y, Takeda T (2008). Nitric oxide production inhibitory activity of flavonoids contained in trunk exudates of *Dalbergia sissoo*. J Nat Prod.

[14] Kanerva L, Estlander T, Alanko K, Jolanki R (2001). Patch test sensitization to compositae mix, sesquiterpene-lactone mix, compositae extracts, laurel leaf, chlorophorin, mansonone A, and dimethoxydalbergione. Am J Contact Dermat.

[15] Rackett SC, Zug K (1997). Contact dermatitis to multiple exotic woods. Am J Contact Dermat.

[16] Beldjoudi N, Mambu L, Labaied M, Grellier P, Ramanitrahasimboia D, Rasoanaivo P, Martin MT, Frappier F (2003). Flavonoids from *Dalbergia louvelii* and their antiplasmodial activity. J Nat Prod.

[17] Chan SC, Chang YS, Wang JP, Chen SC, Kuo SC (1998). Three new flavonoids and antiallergic, anti-inflammatory constituents from the heartwood of *Dalbergia odorifera*. Planta Med.

[18] Kim DC, Lee DS, Ko W, Kim KW, Kim HJ, Yoon CS, Oh H, Kim YC (2018). Heme oxygenase-1-inducing activity of 4-Methoxydalbergione and 4’-Hydroxy-4-methoxydalbergione from *Dalbergia odorifera* and their anti-inflammatory and cytoprotective effects in murine hippocampal and BV2 microglial cell line and primary rat microglial cells. Neurotox Res.

[19] Wang MF, Liu RH, Shao F, Zhang PZ, Mei DY, Yang L, Meng XW (2020). Chemical constituents of *Dalbergia melanoxylon* Heatwood. J Chin Med Mater.

[20] Wu SF, Chang FR, Wang SY, Hwang TL, Lee CL, Chen SL, Wu CC, Wu YC (2011). Anti-inflammatory and cytotoxic neoflavonoids and benzofurans from *Pterocarpus santalinus*. J Nat Prod.

[21] Donnelly DMX, O'Rreilly J, Whalley WB (1975). Neoflavanoids of *Dalbergia melanoxylon*. Phytochemistry.

[22] Donnelly BJ, Donnelly DMX, O'Sullivan AM, Prendergast JP (1969). *Dalbergia* species—VII: The isolation and structure of melanoxin a new dihydrobenzofuran from *Dalbergia melanoxylon guill*. and *perr*. (*leguminoseae*). Tetrahedron.

[23] Panahipour L, Kochergina E, Kreissl A, Haiden N, Gruber R (2019). Milk modulates macrophage polarization in vitro. Cytokine X.

[24] Panahipour L, Kochergina E, Laggner M, Zimmermann M, Mildner M, Ankersmit HJ, Gruber R (2020). Role for lipids secreted by irradiated peripheral blood mononuclear cells in inflammatory resolution in vitro. Int J Mol Sci.

[25] Panahipour L, Nasserzare S, Amer Z, Brücke F, Stähli A, Kreissl A, Haiden N, Gruber R (2019). The anti-inflammatory effect of milk and dairy products on periodontal cells: an in vitro approach. Clin Oral Investig.

[26] Mantovani A, Biswas SK, Galdiero MR, Sica A, Locati M (2013). Macrophage plasticity and polarization in tissue repair and remodelling. J Pathol.

[27] Crascì L, Panico A (2013). Protective effects of many citrus flavonoids on cartilage degradation process. J Biomater Nanobiotechnol.

[28] Im DS (2020). Pro-resolving effect of ginsenosides as an anti-inflammatory mechanism of *Panax ginseng*. Biomolecules.

[29] Naito Y, Takagi T, Higashimura Y (2014). Heme oxygenase-1 and anti-inflammatory M2 macrophages. Arch Biochem Biophys.

[30] Marchesan J, Girnary MS, Jing L, Miao MZ, Zhang S, Sun L, Morelli T, Schoenfisch MH, Inohara N, Offenbacher S, Jiao Y (2018). An experimental murine model to study periodontitis. Nat Protoc.

